# A 0.5–67 GHz Wideband Static 1:2 Frequency Divider in an InP DHBT Technology with Core Transistors Scaling Optimization

**DOI:** 10.3390/mi17060729

**Published:** 2026-06-17

**Authors:** Min Zhang, Qiao Meng, Youtao Zhang, Long Chang, Yi Zhang

**Affiliations:** 1 Institute of RF-&-OE ICs, Southeast University, Nanjing 210096, China; 230198988@seu.edu.cn; 2 Nanjing GuoBo Electronics Co., Ltd., Nanjing 210016, China; 3 National and Local Joint Engineering Laboratory of RF integration & Micro-Assembly Technology, Nanjing 210023, China; 4 Nanjing Electronic Devices Institute, Nanjing 210016, China

**Keywords:** current-mode logic (CML), indium phosphide (InP), static frequency divider, wideband, transistor scaling, millimeter-wave MMIC

## Abstract

This paper presents a 0.5–67 GHz static 1:2 frequency divider implemented in a commercial 0.7 μm InGaAs/InP DHBT technology. Instead of migrating to a more advanced process or introducing complex speed-enhancement circuits, a selective transistor scaling strategy is adopted, where only the critical switching and latching differential pairs in the CML master–slave core are implemented using 0.5 μm high-*f*_T_ DHBTs, while the input/output buffers, bias circuits, and non-critical devices remain based on standard 0.7 μm transistors. This approach reduces the parasitic capacitances at speed-limiting nodes and improves the high-frequency operation of the divider with minimal circuit and process overhead. The fabricated divider achieves a continuous operating bandwidth from 0.5 to 67 GHz, a full-band input power range from −5 to +10 dBm, a single-ended output power higher than −10 dBm, and an SSB phase noise of −141.03 dBc/Hz at 100 kHz offset with a 30 GHz input. These results demonstrate that selective core transistor scaling provides an effective and practical route for upgrading wideband static frequency dividers on mature InP DHBT platforms.

## 1. Introduction

Static frequency dividers are key building blocks in millimeter-wave frequency generation systems. Driven by broadband communication, satellite links, and high-speed instrumentation, they are required to provide wide operating bandwidth, high input frequency, stable phase noise, and sufficient input power tolerance [1,2,3].

Emitter-coupled logic (ECL) and current-mode logic (CML) structures are widely adopted for high-speed static dividers due to their fast switching speed and good noise immunity [4,5,6]. In these dividers, the maximum operating frequency is mainly limited by the switching speed of the master–slave latch core. To extend the operating frequency, circuit-level techniques, such as inductive peaking, asymmetric latch, split-load structures, and *f*_T_-doubler topologies, have been reported [7,8,9,10]. In parallel, InP HBT/DHBT technologies are attractive for millimeter-wave and sub-terahertz dividers due to their high transition frequency and high breakdown voltage, while SiGe BiCMOS offers advantages in power efficiency, integration, and cost [11,12,13].

Despite recent progress, wideband high-frequency division with low implementation overhead remains difficult to achieve. Advanced InP HBT/DHBT technologies can support very high operating frequencies; for instance, a 0.25-μm InP HBT static divider has achieved operation up to 204.8 GHz, but with a DC power consumption of 1820 mW [11]. Circuit-level speed-enhancement techniques, such as *f*_T_-doubler topologies, can further extend the operating range, but usually at the cost of increased latch complexity and layout difficulty [10]. SiGe BiCMOS dividers offer good power efficiency and integration advantages, yet their lower breakdown voltage may restrict the allowable input power range [12]. Meanwhile, mature 0.7-μm InP DHBT platforms provide robust breakdown characteristics and fabrication maturity, but their high-frequency capability still requires improvement. These trade-offs motivate a practical optimization strategy that enhances static divider performance on mature InP DHBT platforms without full process migration or major topology modification.

In this work, such an optimization is achieved by selectively introducing 0.5 μm high-*f*_T_ DHBTs into only the speed-critical switching and latching pairs of a CML master–slave core, while retaining standard 0.7 μm devices in the remaining circuits. This mixed-device strategy reduces core parasitic capacitances and extends the operating bandwidth with limited circuit and process overhead. Based on this approach, a static 1:2 frequency divider is implemented in a commercial 0.7 μm InGaAs/InP DHBT technology, achieving a continuous operating range of 0.5–67 GHz, a full-band input power range of −5 to +10 dBm, and an SSB phase noise of −141.03 dBc/Hz at 100 kHz offset with a 30 GHz input.

## 2. Process and Device Selection

The divider is fabricated on a commercial 0.7 μm InGaAs/InP DHBT process, Nanjing Electronic Devices Institute, Nanjing, China. The DHBT epitaxy is grown by molecular beam epitaxy on an InP substrate, with thin-film resistors, MIM capacitors and multi-layer gold interconnects, for standard 0.7 μm DHBTs, *f*_T_ > 280 GHz, BV_CEO_ > 4 V.

For a DHBT device, the transition frequency can be approximately expressed as:

*f*_T_ ≈ *g*_m_/2π(C_π_ + C_μ_), (1)

where *g*_m_ is the transconductance, *C*_π_ is the base-emitter capacitance and *C*_μ_ is the base-collector capacitance. Transistor scaling reduces parasitic capacitances *C*_π_ and *C*_μ_, thereby increasing *f*_T_. A systematic device-level optimization was performed, which identified 0.5 μm as the optimal emitter length for this process, offering the best trade-off between *f*_T_, current driving capability and process compatibility.

Notably, the 0.5 μm DHBTs are process-compatible variants: only the emitter length is reduced, with all other parameters (epitaxial structure, doping, metal layers, passives) unchanged. No additional fabrication steps are needed. The scaled devices achieve *f*_T_ > 350 GHz while maintaining BV_CEO_ > 4 V, as breakdown voltage depends on epitaxial design, not lateral dimensions. This differs fundamentally from a pure 0.5 μm process, which requires full scaling of all device and interconnect dimensions. While pure 0.5 μm processes reach *f*_T_ > 400 GHz, they have higher wafer cost, doubled cycle times, and lower yield.

The 0.5 μm scaled DHBTs have one primary inherent limitation: lower current driving capability per unit emitter width, requiring 40% wider emitters to deliver the same total current under identical bias conditions. Full-circuit implementation with scaled devices would increase parasitic capacitances and degrade the noise performance of bias circuits.

Thus, a mixed-device strategy is adopted: 0.5 μm high-*f*_T_ devices are used exclusively for speed-critical switching and latching pairs, while standard 0.7 μm DHBTs are retained for I/O buffers, bias circuits and non-critical nodes. This improves core high-frequency performance while retaining full compatibility with the mature 0.7 μm process.

## 3. Circuit Design

**Figure 1:**
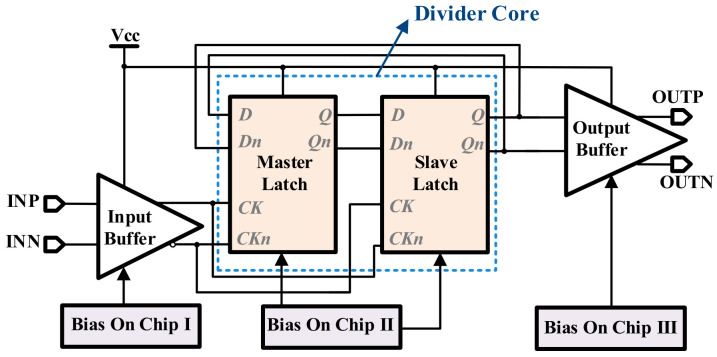
Block diagram of the divider.

The divider consists of a differential input buffer, a CML master–slave divider core, a differential output buffer, and on-chip bias circuits, as shown in Figure 1. Independent biasing is used for the major functional blocks, allowing the input matching, core switching speed, and output drive capability to be optimized separately.

**Figure 2:**
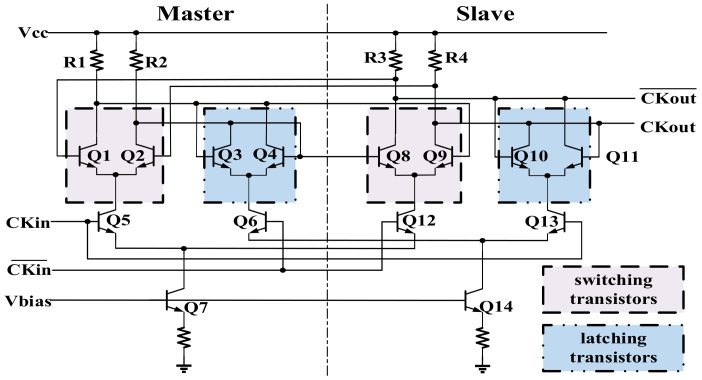
Schematic of the CML master–slave core.

The divider core, shown in Figure 2, adopts a CML master–slave flip-flop topology to realize static 1:2 frequency division. In this topology, the switching and latching differential pairs dominate the maximum toggling speed and therefore form the main high-frequency bottleneck. Accordingly, 0.5 μm × 5 μm high-*f*_T_ DHBTs are used only for the switching pairs Q1/Q2/Q8/Q9 and the latching pairs Q3/Q4/Q10/Q11, while standard 0.7 μm DHBTs are retained for the remaining devices. The 5 μm emitter width was selected through extensive electromagnetic (EM) simulations of all standard 0.5 μm DHBT variants (3 μm, 5 μm, 10 μm, 15 μm emitter widths). A narrower width would limit the maximum switching current, while a wider width would increase parasitic capacitances and degrade high-frequency performance. This selective device allocation reduces parasitic capacitances at the most speed-sensitive nodes without increasing the complexity of the non-critical circuitry. The tail-current network is further optimized to balance switching speed and latch regeneration. The tail transistors Q5/Q12, which control the switching pairs, are designed larger than Q6/Q13, which control the latching pairs, while Q7 and Q14 are sized with a 1:2 ratio. This sizing arrangement is matched to the current density of the 0.5 μm core transistors, ensuring they operate at their peak *f*_T_ point across the entire bandwidth. This increases the available switching current for high-speed operation and maintains sufficient regeneration strength over a wide input-frequency range.

**Figure 3:**
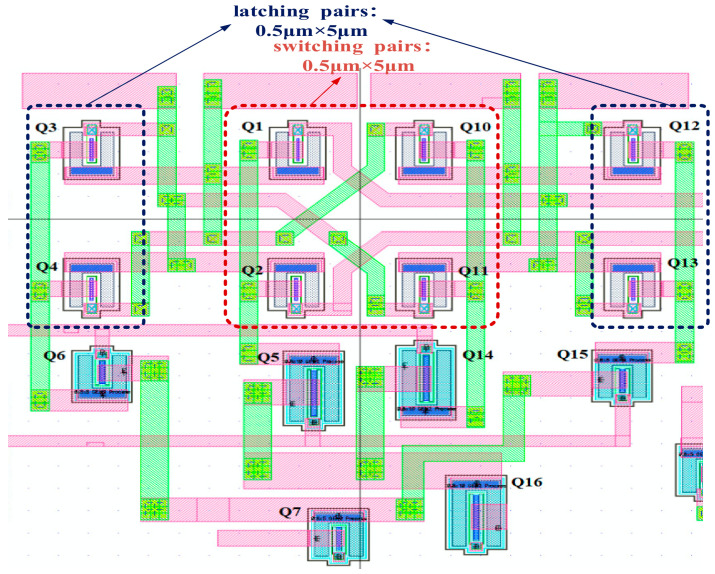
Centrosymmetric layout of the divider core.

To further suppress parasitic imbalance, a fully centrosymmetric (rotational symmetric) layout is adopted for the divider core, as shown in Figure 3. The symmetric placement shortens and balances the differential signal paths, reduces parasitic mismatch, suppresses substrate coupling and common-mode noise, and ensures consistent performance of the master and slave latches at millimeter-wave frequencies.

**Figure 4:**
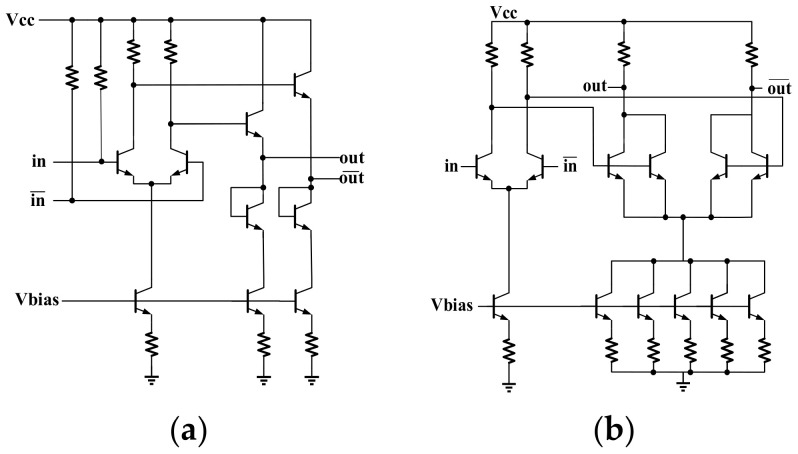
(**a**) Schematic of the differential input buffer; (**b**) schematic of the differential output buffer.

The input buffer, presented in Figure 4a, uses a cascaded differential amplifier followed by an emitter follower to provide wideband input matching and level shifting. The output buffer, shown in Figure 4b, adopts parallel differential stages to achieve sufficient drive strength for off-chip measurement and system integration. The on-chip bias provides stable DC operating points for all blocks. The schematic design and simulation of the proposed frequency divider were performed using Keysight ADS 2021 Update 1, Keysight Technologies, Santa Rosa, California, USA.

## 4. Measured Results

**Figure 5:**
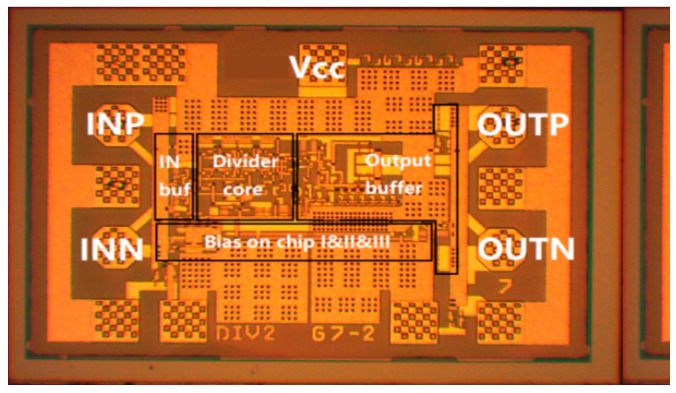
Chip microphotograph.

The chip was fabricated in the 0.7 μm InGaAs/InP DHBT technology, with a die size of 0.88 mm × 0.61 mm, as shown in Figure 5. For measurement, the chip was mounted in a custom-designed metal cavity, and the losses of the measurement setup were calibrated out. The divider operates from a single 3.3 V supply and consumes approximately 350 mW of DC power. The output buffer consumes approximately 120 mW, accounting for 34% of the total power consumption.

**Figure 6:**
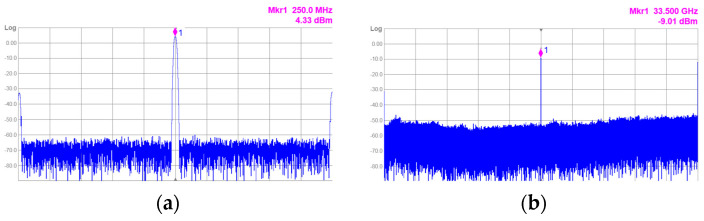
(**a**) Measured output spectrum at 0.5 GHz input signal; (**b**) measured output spectrum at 67 GHz input signal.

**Figure 7:**
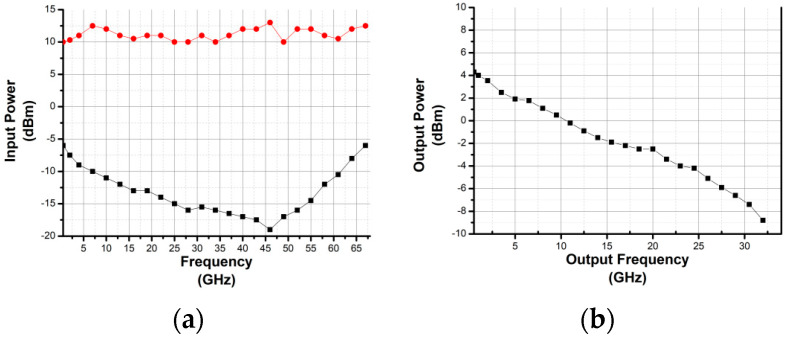
(**a**) Measured input power dynamic range versus input frequency (The red curve with circular markers represents the maximum input locking power of the proposed 1:2 frequency divider, while the black curve with square markers represents the minimum input locking power). The area between the two curves indicates the valid input power range for normal divider operation across the 0.5–67 GHz frequency band. (**b**) measured output power versus output frequency.

Figure 6 shows the measured output spectra at input frequencies of 0.5 GHz and 67 GHz, verifying stable divide-by-two operation at both the low- and high-frequency ends of the operating band. The measured input power range is plotted in Figure 7a. Across the full 0.5–67 GHz band, the divider maintains stable frequency division for input power levels from −5 to +10 dBm, indicating a wide input drive tolerance for broadband operation. The measured single-ended output power versus frequency is shown in Figure 7b. The output power varies from −9 to +4.5 dBm across the operating band and remains above −10 dBm over the full frequency range.

**Figure 8:**
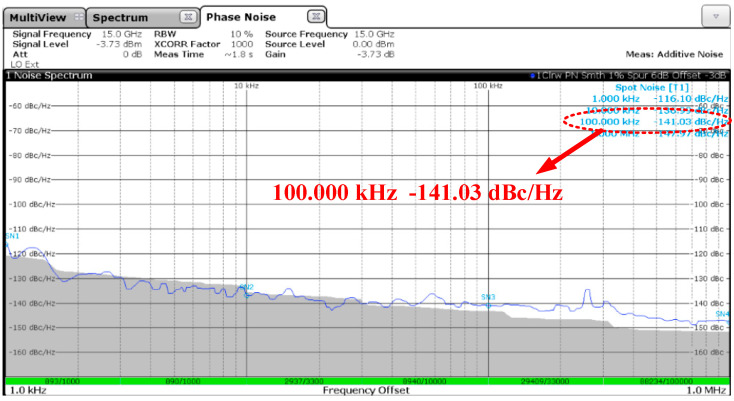
Phase noise at 30 GHz input with 100 kHz offset.

Figure 8 presents the measured single-sideband phase noise with a 30 GHz input. At a 100 kHz offset, the divider achieves an SSB phase noise of −141.03 dBc/Hz. This result indicates that the proposed mixed-device core maintains low-noise operation while extending the high-frequency division range. The input RF signal was provided by an E8257D microwave signal source (Keysight Technologies, Santa Rosa, CA, USA). The output frequency and phase noise characteristics were measured using an FSWP phase noise and and spectrum analyzer (Rohde & Schwarz, Munich, Germany).

Table 1 compares the performance of this work with previously reported state-of-the-art static frequency dividers. A widely used figure of merit (FoM) for static frequency dividers is defined as FoM = BW∙*f*_max_/*P*_DC_ [14]. It should be noted that alternative FoM definitions exist in the literature, such as *f*_max_/*P*_DC_ and SOF/*P*_DC_, which focus primarily on high-frequency performance. For wideband frequency dividers targeting broadband communication systems, the FoM incorporating both bandwidth and maximum frequency provides a more comprehensive characterization of overall performance.

## 5. Discussion

The measured results confirm that selective core transistor scaling based on a standard CML architecture can significantly extend operating bandwidth and increase maximum frequency, achieving a 56% improvement in upper frequency limit compared with our previous work without transistor scaling [1]. This approach also effectively reduces circuit complexity: compared with the *f*_T_-doubler topology in [10], which requires 16 transistors per latch, the proposed CML core only uses seven transistors per latch, significantly simplifying design and layout.

Notably, the proposed design achieves a wider full-band input power range than most reported works, which is a critical advantage for practical system integration. The excellent phase noise performance further enhances its suitability for high-quality local oscillator generation in broadband communication systems.

Although the output power variation is relatively large (−9–+4.5 dBm), it remains well within the input power range of this work. For applications requiring more uniform output power, a two-stage cascaded divider configuration can be employed to flatten the output power response across the full bandwidth.

## 6. Conclusions

A 0.5–67 GHz CML static 1:2 frequency divider is demonstrated in a commercial 0.7 μm InGaAs/InP DHBT technology. By selectively introducing 0.5 μm high-*f*_T_ DHBTs into the speed-critical switching and latching pairs, the mixed-device design improves the high-frequency capability of the divider core without full process migration or major topology modification. The fabricated divider achieves a continuous operating range of 0.5–67 GHz, a full-band input power range of −5 to +10 dBm, single-ended output power above −10 dBm, and an SSB phase noise of −141.03 dBc/Hz at 100 kHz offset with a 30 GHz input. The results verify that selective core transistor scaling provides a practical route for wideband static dividers on mature InP DHBT platforms.

## Figures and Tables

**Table 1:** Wideband Static Divider Performance Comparison.

Ref	[1]	[7]	[10]	[11]	[12]	This Work
Technology	0.7 μm InP DHBT	0.7 μm InP DHBT	0.8 μm InP DHBT	0.25 μm InP HBT	0.18 μm SiGe BiCMOS	0.7 μm InP DHBT (Core: 0.5 μm DHBT)
Transistor *f*_T_ (GHz)	280	280	165	400	280	280/350
*P*_DC_ (mW) ^1^	230	620	417	1820	66	350
Freq. Range (GHz)	0.5–43	1–83	0.2–66	4–204.8	30–100	0.5–67
*f*_max_ (GHz)	43	83	66	204.8	100	67
BW (GHz)	42.5	82	65.8	200.8	70	66.5
SOF	35.2	64	53.9	N/A	92.5	47.7
FoM (GHz^2^/mW)	7.95	10.98	10.41	22.6	106.06	12.73
Phase Noise (dBc/Hz @100 kHz)	−140.72 @26 GHz	−139.4 @7 GHz	−106.5 @30 GHz	N/A	N/A	−141.03 @30 GHz
Output Power ^2^ (dBm)	−3 @20 GHz	−25 * @41.8 GHz	−4.74 @33 GHz	−14 * @26.5 GHz	−51.67 @49 GHz	−9 @33.5 GHz

^1^ All power consumption values represent total DC power including core, bias, and output buffers. ^2^ Output power values marked with “*” are extracted from the output power curves in the corresponding references. N/A: Not available in the reference.

## Data Availability

The original contributions presented in this study are included in the article. Further inquiries can be directed to the corresponding author.
